# Supplementary material for: The role of the T-cell mediated immune response to Cytomegalovirus infection in intrauterine transmission
Source: PLoS One. 2023 Feb 6;18(2):e0281341. doi: 10.1371/journal.pone.0281341 (PMC9901742; doi:10.1371/journal.pone.0281341)
Supplement: Attachment — Submitted filename: Review_PlosOne.pdf [file pone.0281341.s001.pdf]

The manuscript is well written and interesting but has some important issues that should be appropriately addressed in order to be published. There is important information missing in the description of the methodology used. Additionally, the quality of the figures is so low that they are very difficult to decipher. More information is needed in the introduction and in the discussion sections. Please find a detail list of the concerns mentioned:

-Introduction is too succinct, some more detail on the studies mentioned should be given.

-Line 81 introduction: authors say that congenital infection has a prevalence of 0.14-0.7%. In their cohort 24 women out of 60 with primary infection were transmitters (40%). Is there any reason for a high prevalence of congenital CMV infection in this cohort? Could authors discuss on this matter?

-Line 93 introduction: Authors say, "the opposite effect has also been observed". Please rephrase the sentence as studies 8 and 9 do not measure same parameters as studies 3 and 11. Also there is not enough information in some of the studies regarding the stimuli used (CMV lysate, peptides, etc.), so it is not exactly an opposite effect.

-Line 95 introduction: I recommend changing "assess" by "untangle", as the role has been already studied before but there are questions to clarify.

-Line 105 methods: could authors clarify the following? "The analysis of CMV-specific CD4+ T lymphocytes was inferred from the CD8+ T-cell count and the total lymphocyte count".

The gating strategy is missing. A supplementary figure should be done showing the gating strategy for both CD8 and CD4 T cells, including the positive and negative control.

In the methods, please clarify if CD28 and CD49d were added to the negative control (this should have been done to detect spontaneous unspecific stimulation). Please also explain how the response was calculated, was the background from the negative control subtracted?

In general materials and methods section needs to be completed as information regarding some reagents used is missing (providers of CMV peptides and antibodies, clones of antibodies)

-Figures: the quality of the figures is too low. Please ensure that data is visible.

-Line 273 results: "blood VL was determined earlier in pregnancy" it is not clear whether the infection took place earlier or if there was a divergence in the methodology used for sample collection.

-Tables should have a title. Table 1, Some p-values are missing, also Blood viral load data seems to be wrong, please check as it cannot be all zeros and have a significant difference among groups. Table 3, model 1 is only corrected for time from infection, no correction for treatment or response by time was included. Response by time of CD4 T cells should be also analyzed as it was done for CD8 T cells.

-The line numbering of the discussion is missing; this complicates the reviewing process. Authors conclude that there is no association between the CMV-specific T cells response t and fetal transmission. The use of only two CMV peptides rather than the whole ORF should be discussed as a limitation. Also, functional analysis only considered IFNg, while cytotoxicity or TNF production were not assessed, this should be discussed as well as a limitation of the study. The methodological differences among all the studies related to this work should be argued further in the discussion.
