# Supplementary material for: The role of the T-cell mediated immune response to Cytomegalovirus infection in intrauterine transmission
Source: PLoS One. 2023 Feb 6;18(2):e0281341. doi: 10.1371/journal.pone.0281341 (PMC9901742; doi:10.1371/journal.pone.0281341)
Supplement: Attachment — Submitted filename: Review_PONE-D-22-04433.docx [file pone.0281341.s003.docx]

Cytomegalovirus (CMV) is one of the most common viruses associated with congenital infection. CMV infection can be vertically transmitted to the fetus from the mother through the placenta. In this study, Ramos MS et al used a cohort of pregnant women with primary maternal CMV infection to address whether a CMV specific-T cell response in pregnancy lowers the risk of CMV intrauterine transmission. Interestingly, the authors observed no association and suggested that the presence of a detectable viral load in pregnant women with primary CMV is a possible biomarker of CMV fetal transmission. Overall, it is a very timely and important research topic that emphasizes the need for routine CMV screening in pregnant women. However, several key points remain for clarification.

1) The major limitation of the current study is the small size of the clinical cohort which limits the claims drawn from the study. The manuscript in its current state is hard to follow for non-specialists.

2) Are there any significant differences in the blood viral load between women transmitting the virus to the fetus compared to the ones that are non-transmitters?

3) If available the authors should provide information on the T cell exhaustion marker like PD-1 and whether the level of expression differs between transmitters vs non-transmitters.
