# Supplementary material for: The role of the T-cell mediated immune response to Cytomegalovirus infection in intrauterine transmission
Source: PLoS One. 2023 Feb 6;18(2):e0281341. doi: 10.1371/journal.pone.0281341 (PMC9901742; doi:10.1371/journal.pone.0281341)
Supplement: Attachment — Submitted filename: Response to Reviewers.docx [file pone.0281341.s004.docx]

**RESPONSE TO REVIEWERS**

We would like to thank the reviewers for their kind suggestions and comments to the manuscript that will contribute to improve the quality of this study. Our answers to editor and reviewer´s comments can be found below in red color.

**REVIEWERS:**

**Reviewer #2:** Thank you for conducting and presenting this interesting study to further the understanding of CMV transmission during pregnancy. The previous review comments have been addressed satisfactorily with one exception: The data availability statement does not conform to the PLOS requirement. There must be at least one other individual/entity provided to be responsible for ensuring data access.

Data is not publicly available because is protected by European GDPR laws. Data are pseudoanonymized, and PLOS Data Availability policy includes an “exceptions to sharing materials” if “they compromise the privacy or confidentiality of human research subjects”. However, can be formally shared under a formal application and research proposal, after institutional acceptance, and we have included the PI (Dr. Daniel Blázquez-Gamero) as the contact person for the request. We have included the following statement in the methods section.

**Data Availability Statement** (lines 227-231 of the revised manuscript)

Data is not publicly available because is protected by European GDPR. Patient´s data is pseudoanonymized according to GDPR regulation. However, can be formally shared under a formal application and research proposal after institutional acceptance. Please send your proposal to Dr. Daniel Blázquez-Gamero (email: [danielblazquezgamero@gmail.com](mailto:danielblazquezgamero@gmail.com))

There are a few typographical errors in the manuscript, such as no closing parenthesis ")" in multiple places, and some missing or unnecessary punctuation (period and comma). These are very minor, but it would be helpful to readers to have them addressed.

I enjoyed reading this manuscript. Thank you for the opportunity to review.

We have reviewed the manuscript carefully and typographical errors have been amended in the following lines of the revised manuscript:

- Closing parenthesis: lines 142 and 223.
- Deletion of period and coma: lines 223, 255 and 386.

**Reviewer #3:** Cytomegalovirus (CMV) is one of the most common viruses associated with congenital infection. CMV infection can be vertically transmitted to the fetus from the mother through the placenta. In this study, Ramos MS et al used a cohort of pregnant women with primary maternal CMV infection to address whether a CMV specific-T cell response in pregnancy lowers the risk of CMV intrauterine transmission. Interestingly, the authors observed no association and suggested that the presence of a detectable viral load in pregnant women with primary CMV is a possible biomarker of CMV fetal transmission. Overall, it is a very timely and important research topic that emphasizes the need for routine CMV screening in pregnant women. However, several key points remain for clarification.

1) The major limitation of the current study is the small size of the clinical cohort which limits the claims drawn from the study. The manuscript in its current state is hard to follow for non-specialists.
The initial sample size was 135 pregnant women with CMV infection. However, we selected exclusively those with a primary CMV infection (n=60) to better understand the role of time of maternal infection in T cell responses. We are aware that sample size is one of the major limitations of this study and we tried to overcome this point conducting a multicenter and prospective study in 8 tertiary hospitals. However, CMV screening during pregnancy is not mandatory in Spain to date and this is a major limitation for recruitment. We have included a sentence about sample size in Discussion section (lines 401 to 404 of the revised manuscript).

We tried to clarify definitions and explain in detail study design and laboratory methodology in the Methods section, but we are aware that this topic may be difficult for non-specialists.

2) Are there any significant differences in the blood viral load between women transmitting the virus to the fetus compared to the ones that are non-transmitters?
As indicated in table 1, the mean value of blood viral load (VL) was 955 IU/ml (SD ±2730) vs 0 IU/ml in non-transmitters (p=0.231). However, detectable VL was only present in six women, and all of them were transmitters (table 1) (p=0.004). None of the women with an undetectable VL in blood transmitted the infection to the fetus. Those results are included in table 1 and in the Results section (lines 269-270 of the revised mansucript).

3) If available the authors should provide information on the T cell exhaustion marker like PD-1 and whether the level of expression differs between transmitters vs non-transmitters.

Unfortunately, proteins such as PD-1 or other widely expressed proteins on lymphocyte populations, were not assessed in our study.
