# Supplementary material for: The role of the T-cell mediated immune response to Cytomegalovirus infection in intrauterine transmission
Source: PLoS One. 2023 Feb 6;18(2):e0281341. doi: 10.1371/journal.pone.0281341 (PMC9901742; doi:10.1371/journal.pone.0281341)
Supplement: Attachment — Submitted filename: Response to Reviewers.docx [file pone.0281341.s005.docx]

**RESPONSE TO REVIEWERS**

We would like to thank again the reviewers for their kind suggestions and comments to the manuscript, that will contribute to improve the quality of this study. Our answers to editor and reviewer´s comments can be found below in red color.

**JOURNAL REQUIREMENTS:**

Please review your reference list to ensure that it is complete and correct. If you have cited papers that have been retracted, please include the rationale for doing so in the manuscript text, or remove these references and replace them with relevant current references. Any changes to the reference list should be mentioned in the rebuttal letter that accompanies your revised manuscript. If you need to cite a retracted article, indicate the article’s retracted status in the References list and also include a citation and full reference for the retraction notice.

We have reviewed the reference list of the manuscript and its formatting style carefully and have not found any papers that have been retracted or mistakes regarding the style. During the whole review process, we have only added reference number 24 (during the first review in July 2022). That change was already mentioned at that moment, but any other references have been retracted. For an easier finding of references, we have included the DOI number of every paper of the reference list, in addition to traditional volume and page numbers (except for reference number 30, in which no DOI number was available).

**REVIEWERS:**

**Reviewer #2:** Thank you again for this study. I would request one change in the Data Availability statement to comply with the PLOS policy. The contact individual or organization for data requests must be a contact who is not an author of the study. Can the authors please provide the contact information for a Data Access Committee, Ethics Committee, or other body at their institution?

point of contact ensures data will be accessible even if an author changes email addresses, institutions, or becomes unavailable to answer requests.

Thank you for this information. As suggested by the reviewer, we have provided the contact information of the secretary of the Hospital´s Ethics Committee, María Ugalde: e-mail: [mugalde.imas12@h12o.es](mailto:mugalde.imas12@h12o.es). Data can be shared under a formal application and research proposal after institutional acceptance. We have updated the Data Availability Statement (lines 228-231 of the revised manuscript with track changes):

“Data is not publicly available because is protected by European GDPR. However, can be formally shared under a formal application and research proposal after institutional acceptance. Please send your proposal to the secretary of the 12 de Octubre Hospital Ethics Committee: María Ugalde; e-mail: mugalde.imas12@h12o.es”

**Reviewer #3:** The authors have satisfactorily addressed most of my concerns. In particular, the authors have
streamlined the manuscript for broader audience.
